# Brain Barriers and brain fluids research in 2020 and the *fluids and barriers of the CNS* thematic series on advances in in vitro modeling of the bloodbrain barrier and neurovascular unit

**DOI:** 10.1186/s12987-021-00258-z

**Published:** 2021-05-21

**Authors:** Richard F. Keep, Hazel C. Jones, Lester R. Drewes

**Affiliations:** 1 Department of Neurosurgery, University of Michigan, Ann Arbor, MI 48105 USA; 2 Gagle Brook House, Chesterton, Bicester, OX26 1UF UK; 3 Department of Biomedical Sciences, University of Minnesota Medical School Duluth, Duluth, MN 55812 USA; 4 Department of Neurosurgery, University of Michigan, 109 Zina Pitcher Place, Ann Arbor, R5018 BSRB, MI 48109-2200 USA

## Abstract

This editorial discusses advances in brain barrier and brain fluid research in 2020. Topics include: the cerebral endothelium and the neurovascular unit; the choroid plexus; the meninges; cerebrospinal fluid and the glymphatic system; disease states impacting the brain barriers and brain fluids; drug delivery to the brain. This editorial also highlights the recently completed *Fluids Barriers CNS* thematic series entitled, Advances in in vitro modeling of the bloodbrain barrier and neurovascular unit. Such in vitro modeling is progressing rapidly.

Brain barriers and brain fluids research continues to be a vibrant field. For example, over 10,000 articles were published in 2020 (as accessed on Medline/Ovid) on either the bloodbrain barrier (BBB), the brain endothelium, the choroid plexus (CP), cerebrospinal fluid (CSF), brain edema or hydrocephalus. That is too large a body of work to address in this review, but we aim to highlight some of the current themes of such research. As always, the choice of papers to highlight is idiosyncratic, reflecting in part the interests of the editors of *Fluids and Barriers of the CNS*. We apologize for the many important papers that are not cited.

In addition to reviewing the general literature, we also discuss the recently completed *Fluids Barriers CNS* thematic series entitled, Advances in in vitro modeling of the bloodbrain barrier and neurovascular unit. In vitro models are advancing rapidly and providing greater insight into the mechanisms underpinning the BBB and neurovascular unit (NVU) in health and disease.

## Elements of the bloodbrain barriers and the brain fluid systems

### Brain endothelium

Molecular mechanisms regulating BBB function, including tight junctions (TJs), are potential therapeutic targets for a variety of neurological conditions. Sladojevic et al. [1] found that Regulator of G-protein Signaling 5 (RGS5) regulates brain endothelial nitric oxide synthase and TJs in vitro. In vivo, an endothelial specific RGS5 mouse knockout had larger infarcts, worse neurological deficits and more brain edema after stroke, suggesting RGS5 might be a therapeutic target.

Brain endothelial intercellular junctions are complex dynamic structures. To further understand how to manipulate claudin-5, Roudnicky et al. [2] have used human pluripotent stem cell-derived endothelial cells to create a stable cell line expressing claudin5-green fluorescent protein (CLDN5-GFP). They then screened a chemical library and identified 62 compounds that activated CLDN5-GFP. One of which, RepSox, a TGF pathway inhibitor, was further examined and found to stabilize the vasculature and induce the expression of other TJ proteins and transporters. Kakogiannos et al. [3] described an interesting interaction between the TJ proteins, claudin-5 and junctional adhesion molecule A (JAM-A), whereby JAM-A can upregulate claudin-5 expression via the transcription factor C/EBP-alpha. Previously, it was found that JAM-A can also act as a leukocyte adhesion molecule at the BBB [4]. Together, these results suggest important roles for this relatively-understudied brain endothelial TJ protein.

The endothelial cytoskeleton is important for regulating multiple functions and Samus et al. [5] found that the actin-binding protein cortactin may be a therapeutic target in multiple sclerosis. In mouse experimental autoimmune encephalomyelitis (a model of multiple sclerosis), gene inactivation of cortactin reduced neuroinflammation and a lack of cortactin ameliorated leukocyte migration across the brain endothelium in vivo and in vitro. Mehra et al. [6] also reported that activation of N-Methyl-d-Aspartate Receptors (NMDARs) in brain endothelial cells upregulates immune cell infiltration into brain by phosphorylating myosin light chain and subsequent cell shrinkage. Interestingly, endothelial and neuronal NMDARs differ in structure, function and pharmacology.

Age has an enormous impact on the burden of cerebrovascular disease. However, disease mechanisms and treatments are often studied in young animals. Chen et al. [7] examined the effect of normal aging on capillary, arterial and venous brain endothelial cells using single-cell RNA sequencing in mice. They found the biggest changes with age occurred in the capillary endothelial cell transcriptome and that they could be ameliorated by exposure to plasma from young animals, indicating the importance of circulatory factors. Zhao et al. [8] employed a similar approach to examine the effects of aging on the transcriptome of capillary, arterial and venous brain endothelial cells in mice. Aging impacted inflammatory signaling in all segments and particularly impacted energy metabolism and barrier permeability in capillary endothelial cells. The effects of aging could be reversed with a glucagon-like peptide-1 receptor agonist. Yang et al. [9] have identified a switch from ligand-specific receptor-mediated transcytosis at the BBB to a non-specific caveolar transcytosis with aging in mice. That change may be linked to reduced pericyte coverage.

At the other end of the age spectrum, during brain blood vessel development, Chen et al. [10] examined the role of the gene encoding prion protein 2 (*Prnd*), which encodes the protein doppel. Prnd knockout mice had impaired blood vessel morphogenesis, sprouting defects and BBB dysfunction. Similarly, Cottarelli et al. [11] found that fibroblast growth factor binding protein 1 (Fgfbp1) is a novel Wnt/beta-catenin regulated gene and that endothelial cell-specific loss of Fgfbp1 results in transient hypervascularization and a delay in BBB maturation. They found Fgfbp1 concentrates Wnt ligands near endothelial junctions. Interestingly, Veys et al. [12] found that the major brain endothelial glucose transporter, Glut1, is crucial for CNS angiogenesis but not BBB barrier function. Major facilitator superfamily domain-containing 2a (Mfsd2a) is another protein important in BBB development. Wang et al. [13] found that Mfsd2a binds with another protein, Spinster homolog 2, to regulate sphingosine-1-phosphate release from brain endothelial cells which is important for BBB formation and maintenance.

Species differences are a potential confounder for the translation of preclinical data to the clinic. Song et al. [14] compared the transcriptomes of human and mouse brain microvascular endothelial cells and this should provide a valuable resource particularly since much research is mouse based. As noted throughout this review, major advances in our understanding of the brain endothelium and the NVU have utilized transcriptomics and particularly the multiple uses of RNA sequencing (RNA-Seq). Some guidelines on the appropriate approach for performing, analyzing and publishing such studies have recently been published [15].

### Neurovascular unit

The importance of astrocytes in regulating BBB and other cerebrovascular functions has long been recognized (reviewed in [16]). For example, Wnt signaling is important for maintaining the BBB and Guerit et al. [17] recently demonstrated the importance of astrocytic Wnt release in that function in mice. Blocking such release increased BBB permeability, endothelial vesicle formation and brain edema.

Interestingly, Batiuk et al. [18] used single-cell RNA sequencing to identify multiple astrocyte subtypes that vary across and within mouse brain regions raising the possibility of another level of cerebrovascular regulation. Uchida et al. [19] recently described regional differences in transporter expression in brain capillaries from rats and humans. The extent that these changes are related to inherent differences at the endothelium or differences in signals from other cells of the NVU (e.g., astrocytes) merits investigation.

Pericytes are another important component of the NVU: Mae et al. [20] examined the effects of pericyte loss on the cerebral endothelium at the single cell level. They found that pericytes are important for a limited set of BBB functions but have a role in regulating endothelial arterio-venous zonation and angiogenesis. Further evidence on the importance of pericytes in BBB function comes from Gautam et al. [21] who used a pericyte-specific laminin knockout. They found that loss of pericyte laminin caused age-dependent BBB disruption including altered para- and transcellular pathways. Similarly, Sheikh et al. [22] found that neural-specific depletion of members of the non-specific lethal chromatin complex led to a TLR4-mediated inflammatory signaling cascade in neighboring pericytes that in turn led to marked cerebrovascular defects and cerebral hemorrhage. An interesting advance is the description of tunneling nanotubes linking retinal pericytes that have an important role in neurovascular coupling [23].

While the role of astrocytes and pericytes in regulating the BBB has been the subject of extensive investigation, macrophages/microglial cells have received less attention. Ronaldson and Davis [24] recently reviewed the role of microglial cells in BBB regulation. In addition, Delaney et al. [25] found that mutations in colony stimulating factor-1 receptor (Csf-1r) in a rare condition called adult-onset leukoencephalopathy with axonal spheroids and pigmented glia (ALSP), were associated with cerebrovascular pathologies. Csf-1r is critical for macrophage/microglia function and they found that attenuating Csf-1r signaling resulted in remodeling of BBB TJs. Santisteban et al. [26] provided evidence of the importance of brain endothelial cell to perivascular macrophage crosstalk in BBB dysfunction that can occur in hypertension, with endothelial angiotensin II type-1 receptors playing a role in initiating dysfunction but perivascular macrophages being required for a full phenotype.

Similarly, little is known about brain endothelial cell oligodendrocyte interactions. The evidence on those bi-directional interactions and the role of Wnt/-catenin signaling was recently reviewed [27]. While most focus has been on regulation of endothelial cell function by parenchymal cells, there are other examples of brain endothelial cell signals regulating parenchymal cells. For example, endothelial cells in gliomas promote glioma cell migration by secreting extracellular vesicles [28].

Neuronal activity also impacts brain endothelial cell function [29]. Pulido et al. [30] recently identified a core set of brain endothelial genes whose expression is regulated by neuronal activity. Prominent amongst those were efflux transporters. They also found that effects of neuronal activity on the expression of circadian clock genes in the brain endothelium was important in that regulation. Neuronal regulation of brain endothelial function also occurs in areas without a BBB. Thus, Jiang et al. [31] found that melanin-concentrating hormone-expressing neurons regulate the permeability of blood vessels of the median eminence via vascular endothelial growth factor signaling. Another type of BBB regulation can occur with parenchymal cell death. Nishibori et al. [32] review how the nuclear protein High Mobility Group Box-1 (HMGB-1), released after cell injury, acts to induce BBB disruption and neuroinflammation. HMGB-1 is a damage-associated molecular pattern (DAMP) important in brain injury. It and other DAMPs may be therapeutic targets.

As well as regulating brain endothelial function, other elements of the NVU are also directly impacted by disease. Thus, pericytes are prone to HIV-1 infection and Torices et al. [33] found that caveolin-1, occludin and Alix (an early acting endosomal factor) form a complex that regulates infection.

One generally neglected area of barriers research is the blood-nerve barrier. Ubogu [34] and Reinhold and Rittner [35] recently reviewed our current state of knowledge on this barrier. Stubbs [36] discusses the importance of blood-nerve-barrier dysfunction in peripheral neuropathies while Takeshita et al. [37] have developed a human blood-nerve barrier model.

### Choroid plexus

The CP has a circadian rhythm. Thus, Yamaguchi et al. [38] examined the circadian rhythm of CP clock genes and the relationship between the CP rhythm and that in the suprachiasmatic nucleus and the pineal gland. Further, Liska et al. [39] found that the CP circadian rhythm can be reset by circulating glucocorticoids and Furtado et al. [40] found the circadian rhythmicity of the CP clock gene, Bmal1, was disrupted in a mouse Alzheimers disease (AD) model. These findings suggest that CP function is altered during the day-night cycle. This may impact fluid, solute and cell movement across this blood-CSF interface.

In addition to other brain barriers, the CP may be involved in neuroinflammation. Solar et al. [41] have recently reviewed the CP and the blood-CSF barrier in different diseases including those inducing neuroinflammation. Rodriguez-Lorenzo et al. [42] examined inflammatory changes in the CP in multiple sclerosis. While evidence of inflammation was found, they suggest that it plays only a minor role in immune cell infiltration in patients with chronic multiple sclerosis. In contrast, Mottahedin et al. [43] provide evidence of the importance of the CP in neutrophil entry after hypoxiaischemia in neonatal rats and Saul et al. [44] recently described structural and functional alterations at the CP in amyotrophic lateral sclerosis (ALS). Rayasam et al. [45] also found that the CP was an important route for myeloid cell entry after neonatal stroke and that CX3CR1 and CCR2 signaling plays an important role in that infiltration. Nishihara et al. [46] compared the ability of CD4^+^ T helper subsets to cross the BBB and blood-CSF-barrier models in vitro and their results indicate different subsets use alternate routes for migration.

The impact of diet on the CP has received little attention. However, Alimajstorovic et al. [47] recently reported that female rats fed a high fat diet have twice the CSF secretion rate of control diet rats and this may be a mechanism for idiopathic intracranial hypertension that occurs in obese individuals. Obata and Narita [48] reported that a high cholesterol diet or hereditary hyperlipidemia alters CP structure in rabbits.

Our understanding of the role of the CP in health and disease is aided by in vivo imaging measurements in humans. Evans et al. [49] recently described using magnetic resonance imaging (MRI) to measure the movement of water from arterial blood to CSF in human brain as well as mouse. MRI was also used by Zhao et al. [50] to examine CP hemodynamic parameters (flow, transit time) in humans. Eide et al. [51] used MRI to track the clearance of gadobutrol after intrathecal administration in healthy individuals and those with idiopathic normal pressure hydrocephalus. They found a delayed clearance of the tracer by the CP in the hydrocephalus patients. Positron emission tomography (PET) imaging is also being used to study the human CP. Thus, total AV1451 (tau) PET binding to the CP using Gaussian Mixed Model segmentation can distinguish between patients with AD from those with mild cognitive impairment [52].

Another method that may help to advance understanding of the CP is the development by Pellegrini et al. [53] of human induced pluripotent stem cell (iPSC)-derived CP organoids. They already demonstrated fluid secretion and identified multiple functions of different epithelial cellpopulations.

### Meninges

The meninges have long been an understudied tissue. This is gradually changing because of the potential importance of meningeal lymphatics in CSF drainage and a route for immune surveillance of the brain (reviewed in [54]). Interestingly, Haugland et al. [55] recently found that implanting EEG electrodes in mice was enough to induce meningeal lymphangiogenesis and enhance the glymphatic pathway and Chen et al. [56] provided evidence that the meningeal lymphatics play a role in the clearance of red blood cells from CSF after subarachnoid hemorrhage (SAH). They found that ablating the meningeal lymphatics in mice greatly exacerbated SAH-induced brain injury. Being able to track meningeal lymphatic function in patients and how it is impacted by disease would be very useful and Ringstad and Eide [57] are currently using MRI to track the route for tracer movement from CSF to dural lymphatic vessels in humans.

Apart from the dural lymphatic vessels, Shibata-Germanos et al. [58] have identified a cell type they name Leptomeningeal Lymphatic Endothelial Cells (LLECs) within the leptomeninges. These cells have lymphatic and macrophage properties and while they do not form lumens, they phagocytose macromolecules including amyloid- and suggesting they may have homeostatic and immune roles.

Interestingly, Uchida et al. [59] have also stressed the importance of transport at the blood-arachnoid barrier. They found that the total protein expression of several transporters at the arachnoid membrane, including p-glycoprotein and breast cancer resistance protein, was greater than at the CP. The importance of the arachnoid membrane in regulating CSF composition is understudied. Emerging evidence indicates the importance of the meninges as a niche for neural progenitor cells [60]. These cells may be a therapeutic target for treating neurological disorders.

### Glymphatic system

The glymphatic system continues to stimulate much research and many controversies [61] and imaging techniques feature prominently in addressing these issues [62]. In particular, rapid advances in non-invasive MR techniques are promising, but have yet to definitively identify the glymphatic system in humans, and PET imaging is also being developed using radio-labeled tracers [63]. There is considerable interest in the effects of the sleep cycle on the glymphatic system with potential implications for toxin clearance in neurodegenerative diseases such as Alzheimers disease. For example, Hablitz et al. [64] have shown a circadian rhythm in the glymphatic system in mice with a peak glymphatic influx and clearance during the mid-rest phase. Loss of aquaporin-4 abolished the day-night differences. As noted elsewhere in this review, the CP and the BBB also show circadian rhythms. How these systems integrate and impact both toxin and drug clearance is an interesting and important area of future research.

A recent modeling study has emphasized the role of intracranial pressure in determining the relative contribution of the glymphatic system to CSF clearance [65]. Also, Goodman and Iliff [66] highlight the critical importance of maintaining physiological blood gases in glymphatic studies. They found that hypercapnia (as can occur with anesthesia) profoundly reduced both the brain uptake of tracers injected into the subarachnoid space and the appearance of tracers in deep cervical lymph nodes after injection into mouse brain parenchyma.

### CSF analysis

CSF analysis is used in the diagnosis of some neurological diseases [67, 68] although there are concerns over reproducibility [69]. One recent advance is in the use of metagenomic next-generation sequencing (mNGS) of CSF RNA and DNA (reviewed in [70]). It was used to simultaneously screen for a wide range of infectious agents in an un-biased manner.

Many neurological conditions differentially affect women and men. Kamitaki et al. [71] examined the role of differences in the complement system in such sexual dimorphism. A genetic analysis indicated that complement component 4 (C4) genes C4A and C4B contribute to differential risk in systemic lupus erythematosus and Sjogrens syndrome. They also found that CSF concentrations of both C4 and C3 (a downstream effector) were higher in men than women.

One area where CSF biomarkers are increasingly used is in attempting to distinguish types of neurodegenerative disorders. While p-tau181 is an established AD biomarker [67], Janelidze et al. [72] recently reported that p-tau217 may be a more useful biomarker. Similarly, Blennow et al. [73] proposed reductions in a tau fragment, tau368, in CSF as a novel marker of AD as it is sequestered into tangles. It should be noted that particular tau profiles change during AD progression [74]. For vascular dementia patients, Llorens et al. [75] recently found that elevated CSF lipocalin 2 levels can distinguish them from other types of neurodegenerative dementia.

CSF antibodies to glutamic acid decarboxylase (GAD) are found in several neurological conditions. Whether or not these GAD antibodies participate in pathology with GAD autoimmunity was recently reviewed by Graus et al. [76].

Identification of CSF leukocyte populations in disease states has also advanced. Schafflick et al. [77] used single cell transcriptomics to identify CSF leukocyte populations in multiple sclerosis and found that compartmentalized populations were driven by local T cell/B cell interactions. Gate et al. [78] found clonally expanded CD8^+^ T effector memory CD45RA^+^ cells in the CSF of AD patients. These clonally expanded cells had enhanced T cell receptor signaling and had specificity to two separate Epstein-Barr virus antigens.

He et al. [79] have developed an interesting approach for sampling brain lymph fluid from the afferent lymph vessels of deep cervical lymph nodes. A different analyte profile may result from this fluid compared to the CSF sampled from the usual sites (e.g., lumbar puncture).

## Neurological condtions

### SARS-CoV-2/ COVID-19

Neurological symptoms including long-term ones, are common in patients with COVID-19 [80, 81]. The underlying mechanisms for these effects are under intense investigation. There is evidence that the spike protein, S1, of SARS-CoV-2 can cross into mouse brain after IV or intranasal administration [82]. Furthermore, in a few cases of COVID-19 patients with neurological symptoms, SARS-CoV-2 was detected in CSF by PCR [83, 84]. In contrast to a direct parenchymal effect of the virus, it was suggested that antibodies against SARS-CoV-2 cross the bloodbrain barriers and cause the neurological symptoms by an autoimmune-like response [85, 86].

Another possibility is that SARS-CoV-2 may directly affect cells of the NVU and lead to neurological dysfunction [87]. In vitro*,* Buzhdygan et al. [88] found that the spike protein of SARS-CoV-2 induced a loss of brain endothelial cell barrier integrity and triggered a pro-inflammatory response. However, it should be noted that questions have arisen over whether human brain endothelial cells normally express the angiotensin converting enzyme-2 (ACE2) necessary for infection [89]. An alternate barrier site may be the CP and Jacob et al. [90] and Pellegrini et al. [91] have both used human iPSC-derived organoids and found that the virus can infect the CP and disrupt choroid plexus function. Another alternate potential mechanism is that the systemic inflammatory response to SARS-CoV-2 causes secondary effects on the brain [92].

The effect of SARS-CoV-2 is a rapidly evolving area of research. Doubtless, much more will be discovered in 2021 including the impact on neurological function of different SARS-CoV-2 variants.

### Spaceflight

Spaceflight and associated microgravity have emerged as new challenges with respect to the brain and CSF. It has been known for several years that long-duration spaceflight in particular, results in upward shifts in brain tissue, a narrowing of the CSF spaces at the vertex, raised intracranial pressure and increased ventricular volume [93]. The cardiovascular system adapts to weightlessness with an increased cardiac output and accumulation of venous blood in the head [94]. Spaceflight Neuro-ocular syndrome (SANS) is characterized by edema of the optic disc and flattening of the globe with optic nerve tortuosity leading to ophthalmic abnormalities [95]. Changes in CSF hydrostatic gradients and intracranial pressure may be responsible (reviewed in [96]).

### Hydrocephalus

Hydrocephalus is a diverse disorder characterized by enlarged lateral ventricles (ventriculomegaly) with or without increased intracranial pressure. Underlying causes include failure of correct brain development, hypersecretion of CSF, obstructed CSF circulation, insufficient CSF absorption and atrophy of brain tissue.

### Genetic causes for ventriculomegaly

The molecular mechanisms underlying brain ventricular development are still incompletely understood. Yang et al. [97] have identified a member of the neural CAM gene family, Camel, that regulates cell adhesion in zebrafish. Loss of Camel causes hydrocephalus, scoliosis of the spine and failure of the Reissner fiber to form in the ventricular system, whereas increasing Camel mRNA induced Reissner fiber misdirection. Prior results indicated that loss of the Reissner fiber (secreted by the subcommissural organ) can lead to hydrocephalus [98].

Correct cilia function is important for the brain and many examples of defective cilia are associated with hydrocephalus. For example, Robson et al. [99] examined patients with mutations in the *multicilin* gene, MCIDAS, using MRI. They found that all the patients had hydrocephalus, arachnoid cysts, and choroid plexus hyperplasia, possibly related to CSF overproduction. Another cilia protein, Cfap206 is regulated by FOXJ1 and Cfap206 mutant mice develop hydrocephalus, Beckers et al. [100]. Similarly, Zou et al. [101] found that loss of another motile cilia protein, RSPH9, caused hydrocephalus and ependymal cell loss in mice, along with some parenchymal effects. The loss of membrane type 1-matrix metalloproteinase in mice causes a hydrocephalus that is associated with reduced and disorganized motile cilia and altered brain development, (Jiang et al. [102]). Wu et al. [103] found that vacuolar protein sorting associated protein-35 (VPS35) promotes differentiation, survival and ciliogenesis in ependymal cells. It also prevents local microglial cell activation and knock out of VPS35 in ependymal progenitor cells resulted in hydrocephalus.

Clearly, genetic defects are a leading cause for brain to develop abnormally with resultant ventriculomegaly as emphasized by Jin et al. [104] who used whole-exosome sequencing in patients with sporadic congenital hydrocephalus. They found that de novo damaging mutations accounted for~20% of sporadic congenital hydrocephalus cases that required neurosurgical treatment.

### Hydrocephalusother mechanisms

Reduced glycine decarboxylase function in mice and humans with non-ketotic hyperglycinemia is associated with hydrocephalus. Santos et al. [105] found this reflects a defect in folate metabolism and hydrocephalus in glycine decarboxylase deficient mice was prevented by supplementing the maternal diet with formate.

A non-genetic cause of hydrocephalus is intraventricular hemorrhage (IVH). The lysis of red blood cells with the hemorrhage may trigger events contributing to the hydrocephalus. For example, peroxiredoxin 2, the 3rd most common protein in red blood cells, was shown to be a contributor to IVH-induced hydrocephalus in rats and a powerful inflammatory mediator (Tan et al. [106]).

Idiopathic normal pressure hydrocephalus is a condition where CSF circulation and /or absorption may be defective and different MRI techniques are giving improved insight into hydrocephalus and the glymphatic system. Eide et al. [107] used long-term MRI after intrathecal injection of a contrast agent, gadobutrol, to examine CSF tracer dynamics in patients with idiopathic normal pressure hydrocephalus and compared those changes to alterations in CSF system anatomy and neurodegeneration. This may prove a useful tool for examining mechanisms underlying hydrocephalus. Determining which normal pressure hydrocephalus patients will potentially benefit from shunt surgery and which tests are predictive of success continues to be a subject of major concern. For example, Wolfsegger et al. [108] used a quantitative gait analysis scale together with radiological and psychological assessments with CSF tap tests to refine the diagnosis.

### Hydrocephalus treatment

Shunt failure continues to plague hydrocephalus treatment. Work from the Hydrocephalus Clinical Research Network identified factors that may predict fast and ultrafast shunt failure [109]. Age at time of surgery, hydrocephalus etiology and a history of prior failures were important predictors. In contrast, slit or enlarged ventricles were not. A large-scale study of failed shunts (shunt biobank) was developed with the aim of producing a prognostic algorithm [110].

There is a dire need for therapeutics that can alleviate the burden of hydrocephalus. Hochstetler et al. [111] recently reported that antagonists of transient receptor potential vanilloid 4 (TRPV4) channels can ameliorate hydrocephalus in a rat genetic model. Zhang et al. [112] developed a novel, potent SPAK kinase inhibitor that regulates brain cation-Cl cotransporters. Intracerebroventricular administration of the inhibitor reduced CSF hypersecretion in a model of post-hemorrhagic hydrocephalus. It also reduced brain edema and improved outcomes in a model of stroke. The development of treatment strategies may be assisted by in vitro modeling. Castaneyra-Ruiz et al. [113] presented a model for examining the effects of intraventricular hemorrhage on the developing ventricular zone and the associated stem cell niche.

### Dementia

The effects of aging on the brain that lead to cognitive decline and dementia are complex, involving such agents as -amyloid, tau and the susceptibility gene, APOE4. Expression of the latter increases the risk for AD and is associated with earlier onset. Importantly, Montagne et al. [114] recently found that BBB disruption contributes to APOE4-associated cognitive decline in patients independently of AD pathology and that higher levels of ApoE and tau, but not -amyloid and APOE4 gene, were correlated with lower levels of claudin-5 and occludin in AD patient brains (Liu et al. [115]). This suggests there may be multiple NVU targets for reducing the cerebrovascular and cognitive effects of aging. Montagne et al. [114] also found that high CSF levels of a pericyte injury marker predicted cognitive decline in patients that carried the APOE4 gene, but not non-carriers. Furthermore, Blanchard et al. [116], using a human in vitro BBB model, found that APOE4 induces a cerebral amyloid angiopathy-like pathology in pericytes.

Johnson et al. [117] performed a large-scale proteomic analysis of brain and CSF in AD and identified changes in brain glucose metabolism and protein markers associated with an anti-inflammatory state that were also elevated in CSF.

There has been interest in examining changes in the retinal vasculature in AD patients to provide insight into cerebrovascular changes. Shi et al. [118] identified pericyte loss and vascular amyloidosis in AD retina post-mortem and these changes correlated with brain -amyloid burden, cerebral amyloid angiopathy and clinical symptoms. Because -amyloid and tau play a central role in AD pathogenesis there has been great interest in how these molecules are cleared from brain at the bloodbrain barriers and via CSF and the glymphatic system and how such clearance is impacted by the disease itself. Harrison et al. [119] recently reported evidence of impaired glymphatic function and tau clearance in a mouse model of tauopathy, and that such clearance can be affected by an aquaporin-4 inhibitor.

### Stroke and traumatic brain injury

In stroke, there is a question over whether brain endothelial injury is purely a consequence of parenchymal injury or whether it contributes to parenchymal injury. Evidence is accruing, using models with endothelial specific genetic deletions or overexpression, that supports the latter, indicating that brain endothelium is a target for reducing ischemic brain damage. For example, Ma et al. [120] found that endothelial-selective deletion of the microRNA cluster, miR-15a/161, reduced brain infarct, BBB dysfunction and neuroinflammation after stroke in mice. That same deletion increased the expression of claudin-5 and, interestingly, the authors also found that miR-15a/161 binds to the 3 untranslated region of *claudin-5*. Similarly, Sun et al. [121] found that the endothelial-selective deletion of miR-15a/161 also promoted angiogenesis after stroke and improved long-term neurological outcomes in mice. In addition, endothelial specific overexpression of Kruppel-like factor 11 reduced infarct size, BBB disruption, edema and neuroinflammation in mice (Zhang et al. [122]). As noted above, Sladojevic et al. [1] found that mice with an endothelial-specific RGS5 knockout had larger infarcts, worse neurological deficits and more brain edema after stroke. Pericyte dysfunction also plays an important role in stroke pathology. Sun et al. [123] showed that transplantation of human pluripotent stem cell-derived pericyte-like cells improves functional outcomes after stroke in mice.

While severe and moderate traumatic brain injuries are known to cause BBB disruption, the impact of milder injuries is less clear, particularly with repetitive events. OKeefe et al. [124] examined such injury using imaging and blood biomarkers of BBB injury in rugby players and mixed martial arts fighters. They found evidence of BBB dysfunction can occur in a subset of people after repetitive sub-concussive forces. Similarly, Veksler et al. [125] detected BBB dysfunction in American Football players using dynamic contrast-enhanced MRI. They also found BBB dysfunction in rodents exposed to mild repetitive closed-head injury. There is also evidence that acute but mild head trauma causes extravasation from the meningeal vessels into the subarachnoid space [126].

### Brain edema

Brain edema is a major complication in a variety of neurological conditions including stroke, traumatic brain injury and brain tumors and novel treatment strategies and targets are badly needed. Targeting the subcellular distribution of aquaporin 4 (preventing cell surface expression) was found to reduce edema after spinal cord injury in rats (Kitchen et al. [127]). Mestre et al. [128] have proposed the provocative idea that CSF influx into brain parenchyma drives early edema after cerebral ischemia based on evidence that glymphatic influx from CSF to brain is doubled in the early stages of ischemia. Ischemic brain edema is associated with a net accumulation of brain ions and it has been also proposed that ion transport inhibitors may be a method of suppressing edema formation. It is interesting that the SPAK inhibitor developed by Zhang et al. [112] regulates brain cation-Cl cotransporters and reduces ischemic brain edema. It should be noted, however, that it also reduced infarct size which may itself impact edema formation.

### Psychiatric disorders

There has been recent interest in the role of brain endothelial cell/claudin-5 dysfunction in psychiatric disorders, including depression and schizophrenia [129]. Dudek et al. [130] identified factors that may explain vulnerability or resilience of the BBB against the effects of chronic social stress (a model of depression) in mice, effects that also seem to occur in humans. These results may help to identify novel therapeutic strategies for depression. There may be molecular targets within the NVU as a whole as well as specifically the brain endothelium. Thus, Sugimoto et al. [131] reported that serotonin/5HT-1A signaling in the NVU enhances brain endothelial claudin-5 expression and may be linked to altered serotonin signaling found in multiple psychiatric disorders.

Lehmann et al. [132] examined transcriptional changes in the brain endothelial cells of mice exposed to chronic social stress. They found changes related to inflammation, oxidative stress, growth factor signaling and angiogenesis. Interestingly, cessation of the social stress led to a recruitment of leukocytes that may participate in vascular repair. Ouellette et al. [133] also found a vascular component to autism spectrum disorder. Using a mouse model of 16p11.2 deletion autism spectrum disorder syndrome, they found structural and functional cerebrovascular changes and that endothelial cell-specific 16p11.2 deletion recapitulated some of the behavioral changes found in 16p11.2 deletion syndrome.

### Drug delivery

The delivery of therapeutics to the brain for treatment of neurological disorders continues to be a subject for much research. For example, enzyme replacement therapy is being used for the treatment of patients with lysosomal storage disorders. However, such proteins do not cross the NVU/BBB limiting their use in such disorders with CNS involvement and one approach is to use methods to enhance bloodbrain transport. For example, Sun et al. [134] have used nanovesicles of saposin C and dioleoylphophatidylserine to transport -glucosidase into brain in a mouse model of Gaucher disease and found a marked improvement in the neurological phenotype. Also, Hede et al. [135] used a gene therapy approach to examine whether it is possible to induce brain endothelial cells to produce the required protein. For NPC2, the protein that is mutated in Niemann Pick type C2, they have shown, at least in vitro, the feasibilityof such an approach. Interestingly, Gorick et al. [136] showed that pulsed low-pressure focused ultrasound in conjunction with gas-filled microbubbles can be used to transfect the cerebral endothelium without causing BBB disruption.

A more established use of focused ultrasound with microbubbles is to increase the permeability of the NVU/BBB, a technique now in clinical trials. Thus, Rezai et al. [137] demonstrated that it can safely cause transient enhanced permeability in the hippocampus of patients with early AD. Furthermore, DHaese et al. [138] used focused ultrasound-induced BBB/NVU disruption to induce a modest reduction in -amyloid plaque burden in early AD patients.

There continues to be a major focus on targeting receptor-mediated transcytosis at the BBB/NVU for drug delivery. Thus, Kariolis et al. and Ullman et al. [139, 140] used a Fc fragment that targets the transferrin receptor, a receptor that is highly expressed in brain endothelial cells. This fragment has then been used to create antibody transport vehicle molecules for evaluation in mice and monkeys. The Ullmann et al. [140] study demonstrated delivery of iduronate 2-sulfatase to the brain in a mouse model of mucopolysaccharidosis type II, another lysosomal storage disorder, and improvement of brain-related pathology. Stocki et al. [141] also identified single domain antibodies with high affinity for the transferrin receptor, one of which crosses the bloodbrain interface and is taken up by neurons. Intravenous administration of the antibody fused with neurotensin caused a reduction in body temperature (i.e., the construct induced a physiological response).

Georgieva et al. [142] used a human iPSC-based BBB model combined with a human single-chain variable fragment phage display to screen for potential targets for transcytosis. They identified a number of candidates, one of which showed markedly increased uptake into mouse brain. Gregory et al. [143] produced a synthetic protein nanoparticle based on polymerized human serum albumin chemically linked to a cell-penetrating peptide (iRGD). The nanoparticles were loaded with a siRNA against Signal Transducer and Activator of Transcription Factor 3 (STAT3) and given systemically along with ionized radiation to mice with glioblastoma. The treatment resulted in tumor regression and long-term survival in 87.5% of mice. Targeting the cerebral endothelium was also examined by Gonzalez-Carter et al. [144]. They used the low rate of endocytosis in brain endothelial cells to specifically target nanoparticles to the surface of those cells with minimal accumulation in other organs. The cerebral endothelium is capable of undergoing remodeling in disease states (e.g., upregulation of adhesion molecules participating in leukocyte infiltration). Marcos-Contreras et al. [145] used this feature to target the brain in neuroinflammation by creating vascular cell adhesion molecule-1 (VCAM-1) antibody/liposomes as drug carriers.

A novel approach to enhancing drug delivery to the brain was employed by Zhao et al. [146] who used the lymphatic vasculature. They found a subcutaneous injection in the neck close to a lymph node resulted in 44-fold higher drug uptake into brain compared to an intravenous injection.

## New thematic series on advances in in vitro modeling of the BBB and NVU

In vitro models have been very important in understanding the BBB and the NVU. They can provide insights that cannot be obtained by in vivo experiments. However, conventional in vitro models do not fully replicate the BBB/NVU properties found in vivo leading to major efforts to improve the models. *Fluids Barriers of the CNS* published a thematic series aimed at detailing advances in in vitro modeling and providing new insights into brain microvascular endothelial cell (BMEC) and NVU function that have been obtained from in vitro models. Here are some highlights.

A very wide range of in vitro models are now being used to study the BBB/NVU. As reviewed by Bhalerao [147], the field has expanded from static BMEC mono- and co-cultures to include brain organoids, organ-on-a-chip models, spheroids and 3D microfluidic devices. Historically, BMEC primary cultures or BMEC-derived cell lines have been used, but one major advance in the past eight years has been in the use of human induced pluripotent stem cells (iPSCs) to produce BMECs and other cells of the NVU. Unlike conventional BMEC primary cultures and BMEC-derived cell lines, mono- and co-cultures of iPSC-derived BMECs have transendothelial electrical resistances (TEERs) and paracellular permeabilities close to that in vivo [148, 149]. In the thematic series, Workman and Svendsen [150] reviewed recent advances in using such iPSCs to model the BBB in conventional transwell experiments, as well as in 2D microfluidic chips and 3D microvessels.

It should be noted that there are controversies over the phenotype of iPSC-derived BMECs and whether they are endothelial or epithelial. This controversy is addressed by Lippmann et al. in a commentary [151] and they strongly suggest the term BMEC-like be used for these cells. As always, there is a need, where possible, to compare results from such models to in vivo measurements. It is important to benchmark in vitro models [152] and the article of Francisco et al. on the uses of RNAseq in studying the bloodbrain barriers highlights the use of that technique for such benchmarking [15]. With regard to the phenotype of brain endothelial cells, the importance of the microenvironment in vivo for inducing differentiation by signaling pathways (e.g., Wnt/-catenin and others pathways) and dedifferentiation under culture conditions was further characterized [153].

There are still major efforts to improve iPSC-derived BBB/NVU models as shown in the thematic series. One focus has been on the impact of extracellular matrix components. Aoki et al. and Motallebnejad & Azarin [154, 155] both showed the importance of laminin (an important component of the BMEC basement membrane) in enhancing barrier integrity. One major advantage of such human iPSC-derived models is that they can assess the impact of patient-specific genetic mutations on BBB/NVU functions. An example of this is the work of Katt et al. [156] on the effects of genetic mutations on the barrier functions in neurodegenerative disorders.

Cells in the NVU (e.g., astrocytes and pericytes) are also an important determinant of BMEC function. Two papers in this thematic series address the role of pericytes. Jamieson et al. [157] have examined the effect of human iPSC-derived pericytes in 2D and 3D BBB models in vitro and found effects that are model and stress dependent. Heymans et al. [158] have examined the effects of pericyte co-culture on BMEC gene expression, identifying signaling pathways that may underly changes in BMEC function.

Brain endothelial cell tight junctions, transporters and levels of endocytosis/transcytosis are important determinants of BBB function. This thematic series includes studies addressing all those areas in vitro. One use of in vitro models is to examine the mechanisms by which the BBB/NVU is impacted by disease. For example, two are related to stroke: Andjelkovic et al. [159], who review methods of modeling cerebrovascular disease in vitro and Gerhartl et al. [160] who examined the effects of astrocyte and pericyte co-culture on the BMEC response to in vitro ischemia. Neuroinflammation is an important component of many neurological diseases. As reviewed by Erickson et al. [161], the brain endothelium and the NVU play multiple roles in neuroinflammation and in vitro models have enhanced our understanding of those roles. Such models also provide a method for testing potential therapies to limit BBB/NVU dysfunction in disease. Thus, Ge et al. [162] showed the ability of human embryonic stem cell-derived mesenchymal stem cells to repair the BBB dysfunction induced by a major inflammatory mediator, tumor necrosis factor (TNF)-, in vitro.

In summary, the thematic series on advances in in vitro modeling of the BBB and NVU highlights the strides that are being made in the area. In vitro models have helped and will continue to help us gain insights into normal BBB and NVU function as well as the impact of different pathologies.

### Other studies on in vitro modeling of the BBB and NVU

Outside the thematic series, there were important studies on in vitro modeling. A plethora of new models have been developed (too many to review fully here). For example, Nishihara et al. [163] used what they term an extended endothelial culture method for human iPSCs which allows the study of immune cell interactions with BMEC-like cells and Linville et al. [164] have developed a novel 3-D model using human iPSCs to study human brain angiogenesis. Also, brain organoids were made from human embryonic stem cells that form blood vessel-like structures (Ham et al. [165]) and Ahn et al. [166] developed a microphysiological human BBB platform that allows 3D tracking of nanoparticles in the vascular and perivascular spaces.

## Conclusions

We thank the readers, authors, reviewers and editorial board members of *Fluids and Barriers of the CNS*. Despite the impact of COVID-19, 2020 has produced a bumper crop of major advances in the fields of brain barriers and brain fluids research. Thank you all for your contributions.

## Data Availability

Not applicable.
